# Musical hallucination or musical obsession? A differential diagnosis between two cases

**DOI:** 10.1590/1980-5764-DN-2023-0073

**Published:** 2024-06-24

**Authors:** Octavio Pennella Fenelon Costa, Maria Luiza Dalcim, Sumaia Inaty Smaira, Gustavo Bigaton Lovadini

**Affiliations:** 1 Universidade Estadual Paulista, Faculdade de Medicina de Botucatu, Departamento de Neurologia, Psicologia e Psiquiatria, Botucatu SP, Brazil.

**Keywords:** Hallucinations, Obsessive Behavior, Charles Bonnet Syndrome, Obsessive-Compulsive Disorders, Hearing Impairment, Alucinações, Comportamento Obsessivo, Síndrome de Charles Bonnet, Transtornos Obsessivo-Compulsivos, Perda Auditiva

## Abstract

Musical hallucinations and musical obsessions are distinct phenomena. The first can be understood as a manifestation of the musical ear syndrome, which produces deafferentation auditory hallucinations, while the latter is an obsessive symptom of obsessive-compulsive disorders. Both symptoms are often poorly understood and mistaken for one another or for signs of psychotic disorders. We report two cases, one characterized by musical hallucinations and the other by musical obsessions, both with comorbid hearing impairment, which is the main confounding factor in their differential diagnosis. We critically compare the two cases and their key features, allowing diagnostic differentiation and a targeted therapeutic approach.

## RESUMO

Alucinações musicais e obsessões musicais são fenômenos distintos. O primeiro é compreendido como uma manifestação da Síndrome do Ouvido Musical, a qual produz alucinações auditivas por desaferentação, enquanto o último é um sintoma obsessivo dos transtornos obsessivo-compulsivos. Ambos os sintomas são frequentemente pouco compreendidos e confundidos entre si ou com sinais de transtornos psicóticos. Nós relatamos dois casos, um caracterizado por alucinações musicais e o outro por obsessões musicais, ambos com deficiência auditiva comórbida, a qual é o principal fator confundidor no diagnóstico diferencial. Nós comparamos criticamente os dois casos e suas características-chave, permitindo um diagnóstico diferencial e um tratamento direcionado.

## INTRODUCTION

Auditory hallucinations are qualitative changes in perception defined by the conscious experience of sounds that occur in the absence of a corresponding sensory input^1^. Simple auditory hallucinations, such as tinnitus, consist of unformed sounds, such as noises, bells or buzzing, while complex auditory hallucinations are intricate perceptions, in which individuals hear formed sounds, such as music or voices without a corresponding external stimulus^2^. Musical hallucinations (MH) are a subset of complex auditory hallucinations described as hearing music in the form of tunes or melodies, continuously or intermittently, often occurring in individuals unaffected by a psychopathological disorder and with preserved insight^3^. The phenomenon may occur in individuals with acquired hearing loss and normal cognitive function, characterizing the nosological entity known as musical ear syndrome (MES)^4^.

In the 1990s the phenomenon known as Charles Bonnet syndrome, described by visual hallucinations in visually impaired patients with preserved insight, was found out to be much more common than previously thought^5^. Recently, Linszen et al.^6^ outlined that its analogous phenomenon in the auditory sphere, MES, is also more prevalent than previously thought, with 16.2% of individuals with acquired hearing loss experiencing MH, rising to 24% in those with severe impairment. To diagnose MES, it is necessary to exclude pre-existing neuropsychiatric conditions that best explain the origin of the hallucinations, such as depression, schizophrenia, Parkinson's disease, global cortical atrophy, brain lesions (cerebrovascular disease, demyelinating diseases, tumors), temporal lobe epilepsy, infectious encephalitis processes, and substance withdrawal syndromes^2,7^.

Chronic hearing impairment leads to a process of deafferentation of the auditory tracts^8^, which would generate spontaneous activity in auditory pathways, that is, the perception of sounds without external stimuli triggering it^9^. This is the most accepted hypotheses regarding the pathophysiology of MES^10^. Deafferentation might be potentiated by the reduction of auditory distraction, enabling fragments of auditory memory to reach consciousness more easily in silent environments^11^.

Musical obsessions (MO) are a distinct phenomenon from MH, being more common in the context of obsessive-compulsive disorder (OCD), characterized by psychic distress resulting from dysfunctional interpretations of experienced "intrusive musical imagery" (IMI)^12^. IMI are commonly known as "earworms", a non-clinical condition of unintentionally reviving the auditory image of a tune, without deliberate effort and often in repetition, occurring from several times a day to monthly^13^. IMI occurs in about 90% of the general population, being mostly perceived as pleasant or neutral, and often successfully manageable when perceived as annoying or unpleasant^14^. According to Wahl et al.^15^, the lower the agreement with the dysfunctional interpretations, the stronger the positive relationship between frequency and severity of recurrent IMI, therefore increasing the chance of these phenomena becoming obsessive and thus classifiable as MO. This occurs in 1% of the general population and in 5% of the population diagnosed with OCD, fulfilling criteria for obsessions according to the Diagnostic and Statistical Manual of Mental Disorders (DSM-V)^15^.

We report two cases involving musical symptomatology in patients with acquired hearing loss, the first being unaffected by other mental disorders and the second with a comorbid OCD.

## CASE REPORT

### Case 1

An 81-year-old woman with five years of formal education, a retired telephone operator, with a history of bilateral sensorineural hearing loss for the previous five years, sought psychiatric consultation due to intense psychic distress for the previous three months related to a complaint of hearing a child crying. She perceived the sounds all day long, worsening at night, experiencing significant emotional distress, which prompted her to investigate the origin of the sounds throughout her neighborhood. Two years earlier, she had started hearing religious hymns and folk music that nobody else heard, believing it originated from the surroundings. She was familiar with all these melodies, recognizing them as common during her youth. All the reported abnormal sounds disappeared while the patient watched television or slept, returning afterwards. The use of her hearing aid did not suppress any of the reported sounds. During psychiatric evaluation, we detected no depressive, manic, anxious, or obsessive symptoms. There were no delusional constructions linked to the hallucinations, and the patient did not suffer impairments over her basic or instrumental daily activities due to the symptoms. She denied use of alcohol and psychoactive drugs. Physical and neurological examinations were normal.

The patient had a previous history of colon adenocarcinoma treated seven years earlier with surgery and chemotherapy, chronic pain, systemic arterial hypertension and dyslipidemia. She reported regular use of pregabalin 75 mg, duloxetine 60 mg, losartan 50 mg, simvastatin 40 mg, and vitamin D 2,000 UI. The patient's previous assistant mistakenly diagnosed her with psychotic depression and attempted treatment with quetiapine 25 mg and sertraline 100 mg, with no effect over the reported symptoms.

The Mini-Mental State Examination (MMSE) scored 27, within the normal range for her level of education. The Hamilton Depression Rating Scale (HDRS-17) scored 4, and the Geriatric Depression Scale scored below the cutoff. The Edmonton Frail Scale scored 4 (not frail). The Hopkins Verbal Learning Test - Revised, Rey complex figure test, and semantic verbal fluency (animals) test excluded cognitive impairments in the tested domains. The brain computed tomography scan revealed carotid atheromatosis, but without severity criteria. The laboratory tests performed were unremarkable. The audiometry confirmed bilateral moderate/severe sensorineural hearing loss. We diagnosed the patient with probable MES.

As a first intervention, we switched quetiapine to risperidone 1 mg/day and gradually suspended sertraline, without any improvement over her symptoms. Since the patient complained of postural instability and daytime sedation, we suspended the risperidone soon afterwards. Based on a literature review article, we introduced the cholinesterase inhibitor donepezil 5 mg/day^16^, with observed relief over her psychic distress and ceasing of the child's crying. However, the church hymns and folk music continued at the same volume and pattern as before. We adjusted donepezil to 10 mg/day, but without further response. Attempted associations with Carbamazepine 400 mg/day and Aripiprazole 5 mg failed to obtain remission of the musical symptoms, with adverse reactions prompting the medications’ suspension. Nonetheless, the patient stopped searching her neighborhood for explanations and reported being minimally bothered by the music.

### Case 2

A 66-year-old woman, with four years of schooling, a homemaker, with a history of bilateral neurosensory hearing loss for the previous 12 years, sought psychiatric consultation due to intense psychic distress related to hearing religious chants for the previous seven years. She perceived an increase in the religious chant's volume and frequency as her concerns over her family worsened, attempting to suppress them through compulsive religious rituals (prayers). The musical symptoms worsened when the patient's hearing aid was unused. She reported that the music and sounds repeated as a manner of spiritual punishment for all past tragic outcomes in her family members’ lives, of which she deemed herself responsible. When logically challenged, the patient comprehended that her interpretations were dysfunctional and exaggerated, and that the sounds were a product of her own mind. She obsessively complained about the possibility of accidents happening to her grandchildren, exaggeratedly checking their whereabouts, dialing excessively and inappropriately, and looking for them in dangerous neighborhoods. Since her early 20s, when her children were under kindergarten, the patient already suffered from the mentioned obsessive-compulsive symptoms, although without experiencing any musical symptoms. She later explained these symptoms did not concern her enough to warrant medical evaluation and did not imagine that they might have been linked to the musical symptoms that brought her to our service. Upon psychiatric evaluation, we excluded any depressive, dissociative-conversive, psychotic or delusional signs or symptoms. She denied use of alcohol and psychoactive drugs. Physical and neurological examinations were normal.

As comorbidities, the patient reported systemic arterial hypertension, type 2 diabetes, dyslipidemia, hypothyroidism, asymptomatic hyperuricemia, and osteoporosis. Her clinical prescription consisted of enalapril 40 mg, metformin 500 mg, simvastatin 20 mg, levothyroxine 100 mcg, allopurinol 200 mg, vitamin D 1,000 UI, and sodium risedronate 35 mg. The patient's former assistant attempted treatment with sertraline 150 mg/day and risperidone 2 mg/day, with partial improvement over both aggressive obsessions and checking compulsions. However, the disturbing religious chants reported had not ceased, and their content remained unaltered.

The MMSE scored 28, within the normal range for her level of education. The HDRS-17 scored 6, while the Yale-Brown Obsessive-Compulsive Scale (Y-BOCS) scored 19 (10 for obsessions and 9 for compulsions). Audiometry showed moderate mixed hearing loss in the right ear and severe hearing loss in the left ear, suggestive of bilateral otosclerosis. Brain magnetic resonance imaging (MRI) revealed an empty sella turcica, microangiopathy (Fazekas 1) and residual calcifications, without hormonal repercussions or severity. Both electroencephalogram (EEG) and laboratory tests performed were unremarkable. We diagnosed the patient with probable MO within the context of OCD.

As a first intervention, we replaced sertraline with fluvoxamine 50 mg/day, resulting in improvement in the intensity of the reported religious chants and the associated psychic distress. We further increased fluvoxamine to 100 mg/day, resulting in the disappearance of the musical symptoms. On reapplication of the Y-BOCS scale, the patient scored 10 (6 in obsessions and 4 in compulsions), indicating global improvement on her OCD. We opted to maintain risperidone at 2 mg/day, for its contribution over OCD treatment and the patient lacking associated adverse reactions.

## DISCUSSION

In case 1, we observed that the patient experienced the symptoms as concrete and real perceptions, located in objective space, independently of her will, consistent with previous characterizations of MH^17^. The reported content of the hallucinations consisted of melodies meaningful to the patient, which had been previously observed^6^. The patient's insight over the symptoms was initially absent, which could mislead diagnosis in direction of psychotic disorders; however we did not observe any other symptoms that might justify it, such as delusions, negative symptoms of schizophrenia, melancholia, anhedonia. We also excluded incipient dementia since the patient scored well on our cognitive tests and did not present neurological symptoms. Therefore, the MH are better explained by a deafferentation process in the context of MES^9^, further justified by the quick partial response with the cholinesterase inhibitor treatment^16^.

In a manner distinct from case 1, in case 2 we observed that the patient experienced the symptoms as intrusive and repetitive, with associated compulsions and resistance attempts, developing subjective explanations for the phenomenon within an obsessive context of guilt, doubts and magical thoughts, and had from the beginning the insight that the perceptions originated in her own mind, without signs of delusions. Such phenomenology is more suggestive of MO than it is of MH^17^. Considering the patient's comorbid OCD and successful treatment with the selective serotonin reuptake inhibitor (SSRI) fluvoxamine and the atypical antipsychotic risperidone, the diagnosis of musical obsessions becomes likelier^18^.

Neglect and underdiagnosis are common both with MH^3^ and MO^12^, and are thus subject to being misdiagnosed with one another or with psychotic disorders^10^. Some traits shared by our patients contribute to biasing both diagnostic investigations towards MH and MES. Acquired hearing impairment is an important risk factor for developing MH, but not MO, being the main confounding comorbidity in our cases. Female sex, while associated with MH, is not linked to MO, while the age of both our patients is closer to the mean of MH (59 years old) than of MO (33 years old)^6,12^. Despite these misleading traits, our patients had key psychopathological and phenomenological distinctions that set them aside, which allowed us to treat them effectively.

Some non-pharmacological treatments can be effective for both MH and MO. Sound distraction, such as pleasant ambient sounds, television, and radio, can attenuate MH^19^, while people experience IMI less frequently when they are successfully engaged in challenging cognitive tasks, such as verbal or non-verbal puzzles^20^. Counseling and education over the benignity of MH may help patients manage them^21^, while cognitive behavior therapy is an effective option to treat MO^22^. Auditory rehabilitation, while being indicated for tinnitus, has inconsistent impacts over MH in the context of MES^23^. We found no mention of such treatment for MO. Table 1 compares the key aspects of both MH and MO.

**Table 1:** Comparison between musical hallucinations (case 1) and musical obsessions (case 2).

Criteria	Musical hallucinations	Musical obsessions
Phenomenology	Situated in objective space, concrete, independent of will	Situated in figurative space, subjective, has associated resistance and/or compulsions
Insight	With or without insight	Preserved insight
Etiology	Musical ear syndrome	Obsessive-compulsive disorder, obsessive-compulsive personality disorder
Pharmacological treatment	Cholinesterase inhibitor	Selective serotonin reuptake inhibitor, Atypical antipsychotic
Therapeutic interventions	Counseling and education	Cognitive Behavior Therapy
Distraction	May improve symptoms	May improve symptoms
Hearing impairment adaptations	Inconsistent effect over symptoms	Uncertain effect over symptoms

In conclusion, to our knowledge, this is the first case report comparing MHs with MOs, which are distinct entities. While the first are a manifestation of the musical ear syndrome, the latter are a symptom of obsessive disorders. Hearing impairment is an important confounding factor that biases the diagnostic investigation towards musical ear syndrome. In this study we aimed to highlight similarities and differences between our cases to ease future understanding of both conditions and to avoid misdiagnosis with other psychiatric disorders, mainly psychotic conditions.
